# 293. Faecal microbiota transplant to ERadicate gastrointestinal carriage of Antibiotic Resistant Organism: a prospective, randomised placebo-controlled feasibility trial - The FERARO study (IRCTN reg. no. 34467677)

**DOI:** 10.1093/ofid/ofad500.365

**Published:** 2023-11-27

**Authors:** Blair Merrick, Désirée Prossomariti, Michael Kertanegara, Elizabeth Allen, Chrysi Sergaki, Adrien D Le Guennec, Marc Delord, Maria R Conte, David Moyes, Manu Shankar-Hari, Abdel Douiri, Anna L Goodman, Debbie L Shawcross, Simon D Goldenberg

**Affiliations:** Guy's and St Thomas' NHS Foundation Trust and King's College, London, London, England, United Kingdom; Guy's and St Thomas' NHS Foundation Trust, London, England, United Kingdom; Guy's and St Thomas' NHS Foundation Trust, London, England, United Kingdom; Guy's and St Thomas' NHS Foundation Trust and IQVIA, London, England, United Kingdom; Medicines and Healthcare products Regulatory Agency, London, England, United Kingdom; King's College, London, London, England, United Kingdom; King's College, London, London, England, United Kingdom; King's College, London, London, England, United Kingdom; King's College, London, London, England, United Kingdom; University of Edinburgh, Edinburgh, Scotland, United Kingdom; King's College, London, London, England, United Kingdom; Guy's and St Thomas' NHS Foundation Trust and University College, London, London, England, United Kingdom; King's College Hospital NHS Foundation Trust and King's College, London, London, England, United Kingdom; Guy's and St Thomas' NHS Foundation Trust and King's College, London, London, England, United Kingdom

## Abstract

**Background:**

The gastrointestinal tract (GIT) is a reservoir of antibiotic resistant organisms (ARO). ARO colonisation precedes infections which are more challenging to treat than if drug susceptible, with excess morbidity and mortality. Presently, no effective GI decolonisation strategy exists. Faecal microbiota transplant (FMT) has emerged as a potential therapeutic. However, there is uncertainty about feasibility, safety and effectiveness.

**Methods:**

**Population:** Patients with history of invasive infection with extended spectrum beta-lactamase (ESBL) or carbapenemase producing Enterobacterales (CPE) and evidence of persistent GIT ARO carriage (assessed by culture)

**Intervention:**

3 doses of encapsulated lyophilised FMT each manufactured from 80g raw stool given on consecutive days

**Comparator:**

Matched placebo capsules

**Outcomes:**

Primary outcome - participant consent rate (as proportion of those approached screened for GIT carriage of ESBL-E/CPE). Secondary outcomes – safety and efficacy metrics including adverse event (AE) rates and GIT ARO carriage rates at 1 week, 1, 3 and 6 months. Exploratory outcomes – metagenomic and metabolomic stool analyses.

**Results:**

Of 460 approached individuals, 124 (27%) consented. 53/124 participants (43%) fulfilled eligibility criteria. 44/53 (83%) randomised and 41/44 (93%) received investigational medicinal product (IMP): 20 FMT and 21 placebo. 39/41 (95%) completed dosing, 18/20 (90%) FMT (1 investigator and 1 participant discontinuation) and 21/21 (100%) placebo. Overall, GIT AE including reflux, bloating, change in bowel habit, were more common in FMT arm. 6 serious AE in 5 participants occurred in FMT arm, compared to 1 in placebo (all hospitalisation); none were deemed attributable to FMT. No unanticipated harms from FMT administration were realised. GIT ARO carriage rate assessed in 38/41 (93%) IMP recipients providing stool for analysis at 1-month was lower in FMT compared to placebo - FMT: 13/18 (72%) vs. placebo: 17/20 (85%), p=0.34.

FERARO trial consort diagram

**Figure:**
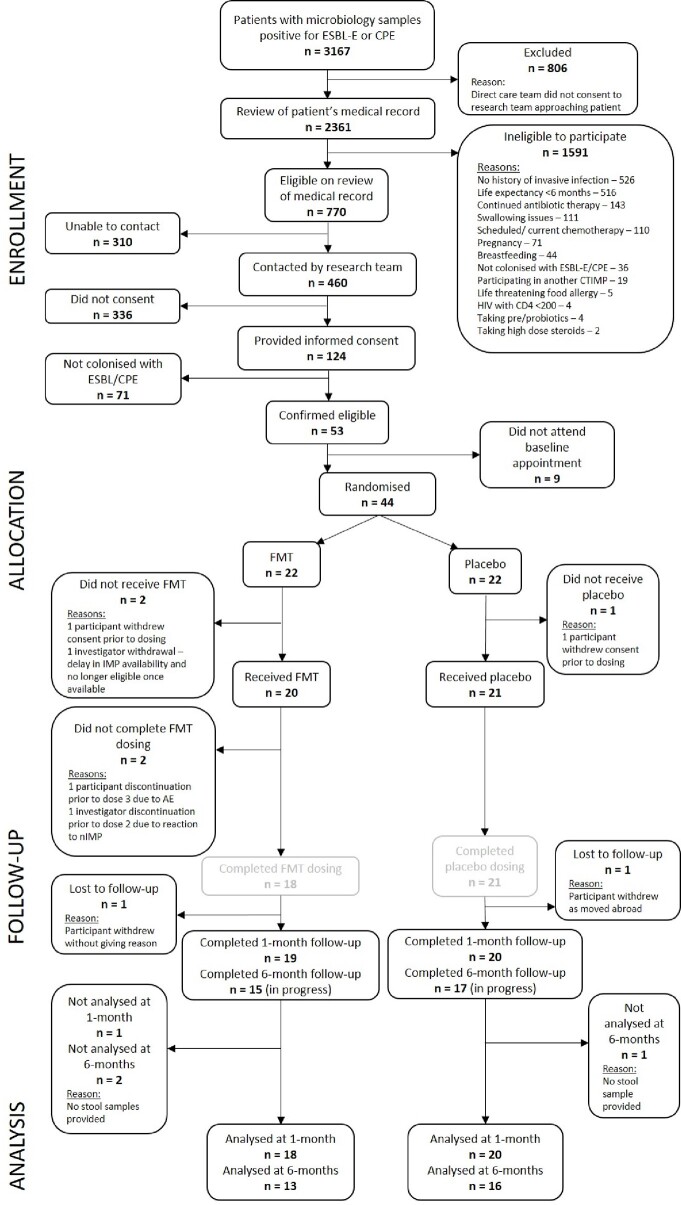

ESBL/CPE detection rate

**Figure:**
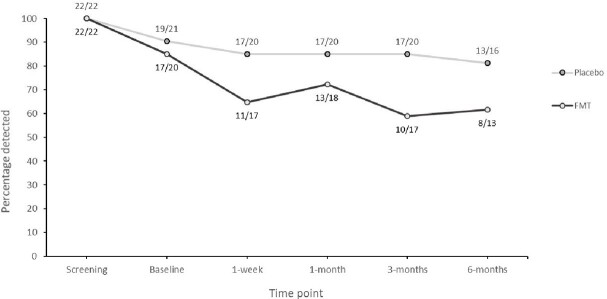

Detected by culture over time

**Conclusion:**

Based on a priori assessment, a substantive trial would be feasible with protocol modifications. Encapsulated FMT appears safe intervention for eradication of GIT ARO carriage. Our feasibility trial informs design, and provides evidence to conduct future efficacy randomised controlled trials.

**Disclosures:**

**David Moyes, PhD, MSc, BSc**, Pfizer: Grant/Research Support **Simon D. Goldenberg, FRCPath, MD (Res), PGDipID, DipHIC, MSc**, EnteroBiotix: Honoraria|Tillotts Pharma UK: Honoraria

